# Supplementary material for: Genomic epidemiology and phenotypic characterisation of Salmonella enterica serovar Panama in Victoria, Australia
Source: PLoS Negl Trop Dis. 2024 Nov 20;18(11):e0012666. doi: 10.1371/journal.pntd.0012666 (PMC11616866; doi:10.1371/journal.pntd.0012666)
Supplement: S1 Fig — (PDF) [file pntd.0012666.s005.pdf]

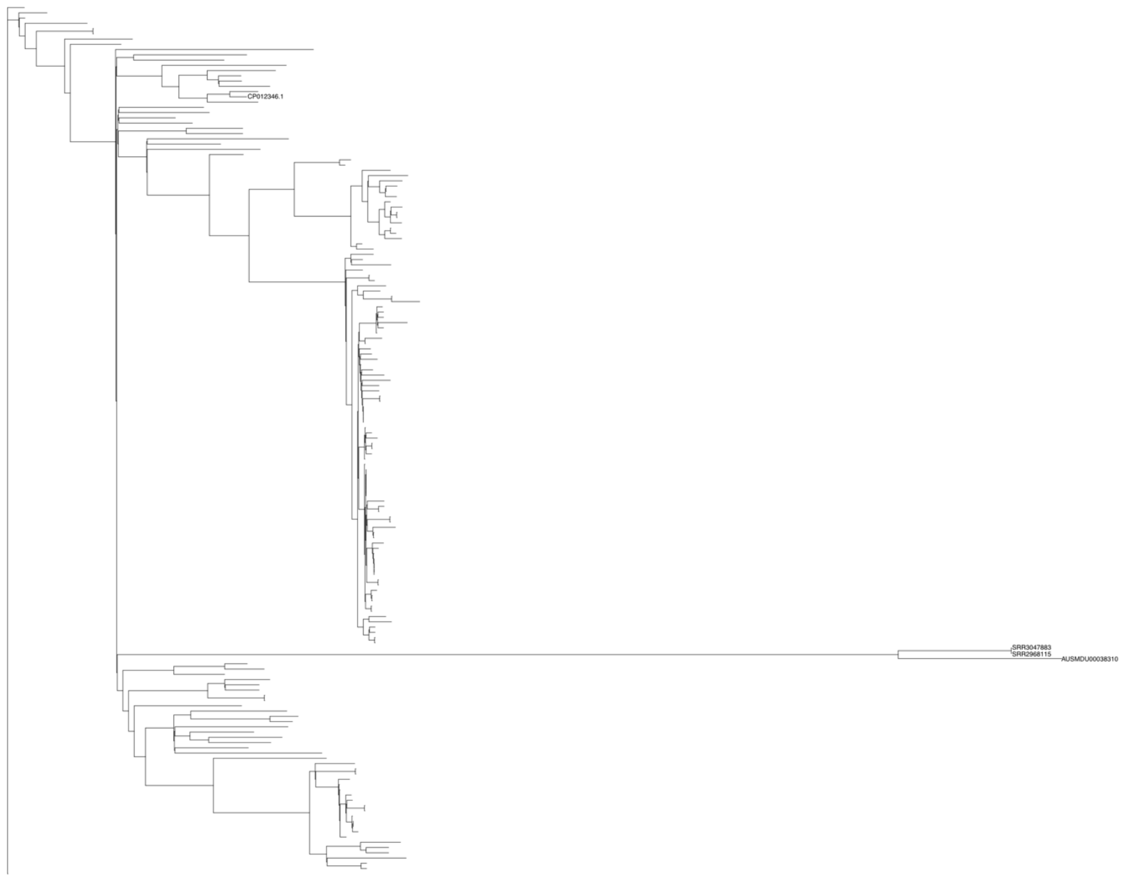

Supplementary Figure 1: Unrooted global maximum likelihood phylogeny of all isolates

The phylogeny was inferred from a total of 165 isolates including 90 Australian and 75 publicly available isolates, with CP012346 as the reference strain. Three isolates on the long branch, AUSMDU00038310, SRR3047883 and SRR2968115 were excluded from subsequent analyses.
