# Supplementary material for: Genomic epidemiology and phenotypic characterisation of Salmonella enterica serovar Panama in Victoria, Australia
Source: PLoS Negl Trop Dis. 2024 Nov 20;18(11):e0012666. doi: 10.1371/journal.pntd.0012666 (PMC11616866; doi:10.1371/journal.pntd.0012666)
Supplement: S2 Fig — (PDF) [file pntd.0012666.s006.pdf]

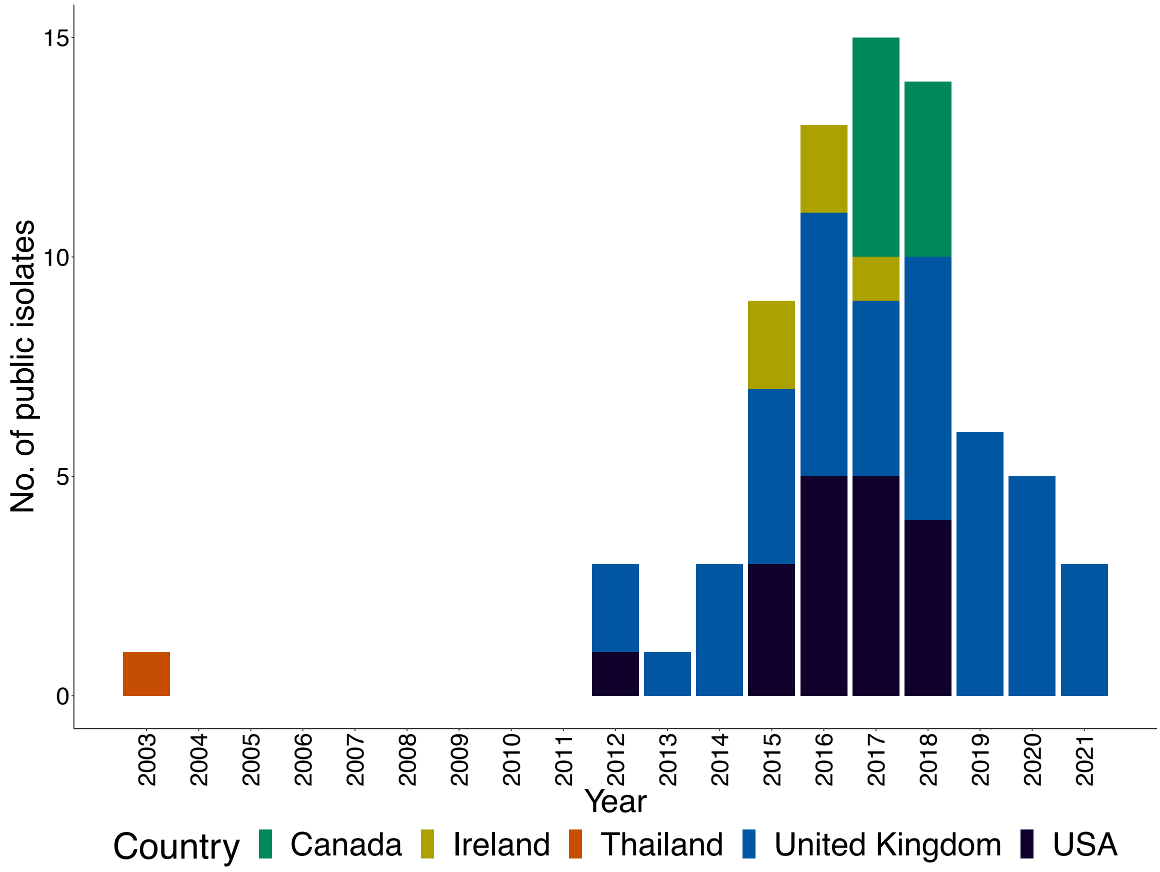

Supplementary Figure 2: Reported year of collection and geographical site of the publicly available isolates included in the study.

The stacked bars indicate total number of isolates that were sampled from each year from the different countries designated by the colours.
