# Supplementary material for: Genomic epidemiology and phenotypic characterisation of Salmonella enterica serovar Panama in Victoria, Australia
Source: PLoS Negl Trop Dis. 2024 Nov 20;18(11):e0012666. doi: 10.1371/journal.pntd.0012666 (PMC11616866; doi:10.1371/journal.pntd.0012666)
Supplement: S3 Fig — (PDF) [file pntd.0012666.s007.pdf]

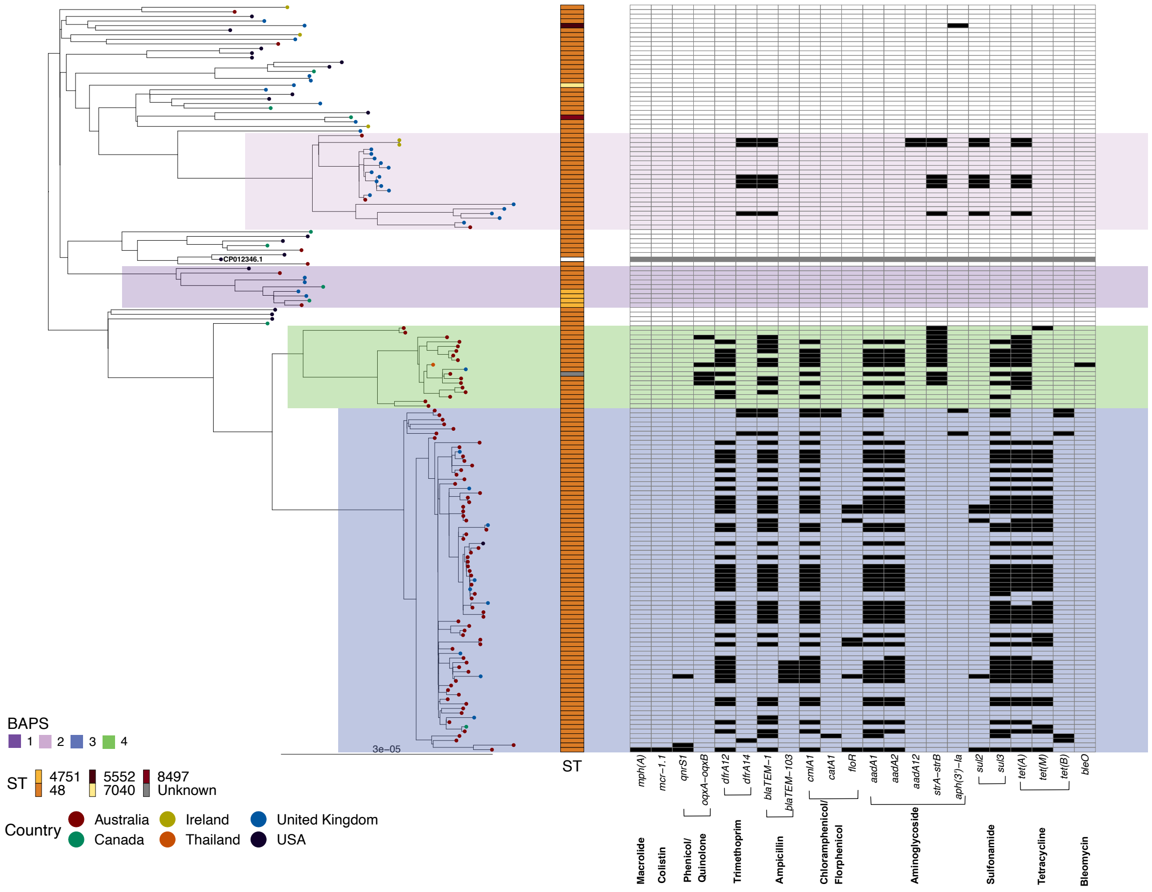

Supplementary Figure 3: Global ML phylogeny showing the ST and AMR gene distribution

The phylogeny was inferred from 162 isolates including 89 Australian and 73 publicly available isolates, with CP012346 as the reference strain. The highlighted clades indicate BAPS lineages. Tree tips are coloured by country of isolation of the samples. The sequence type (ST) are shown to the right of the tree. The AMR heatmap shows the genes detected in AbritAMR for the complete dataset. Scale indicates SNPs substitutions per unit branch length.
