# Supplementary material for: Genomic epidemiology and phenotypic characterisation of Salmonella enterica serovar Panama in Victoria, Australia
Source: PLoS Negl Trop Dis. 2024 Nov 20;18(11):e0012666. doi: 10.1371/journal.pntd.0012666 (PMC11616866; doi:10.1371/journal.pntd.0012666)
Supplement: S4 Fig — (PDF) [file pntd.0012666.s008.pdf]

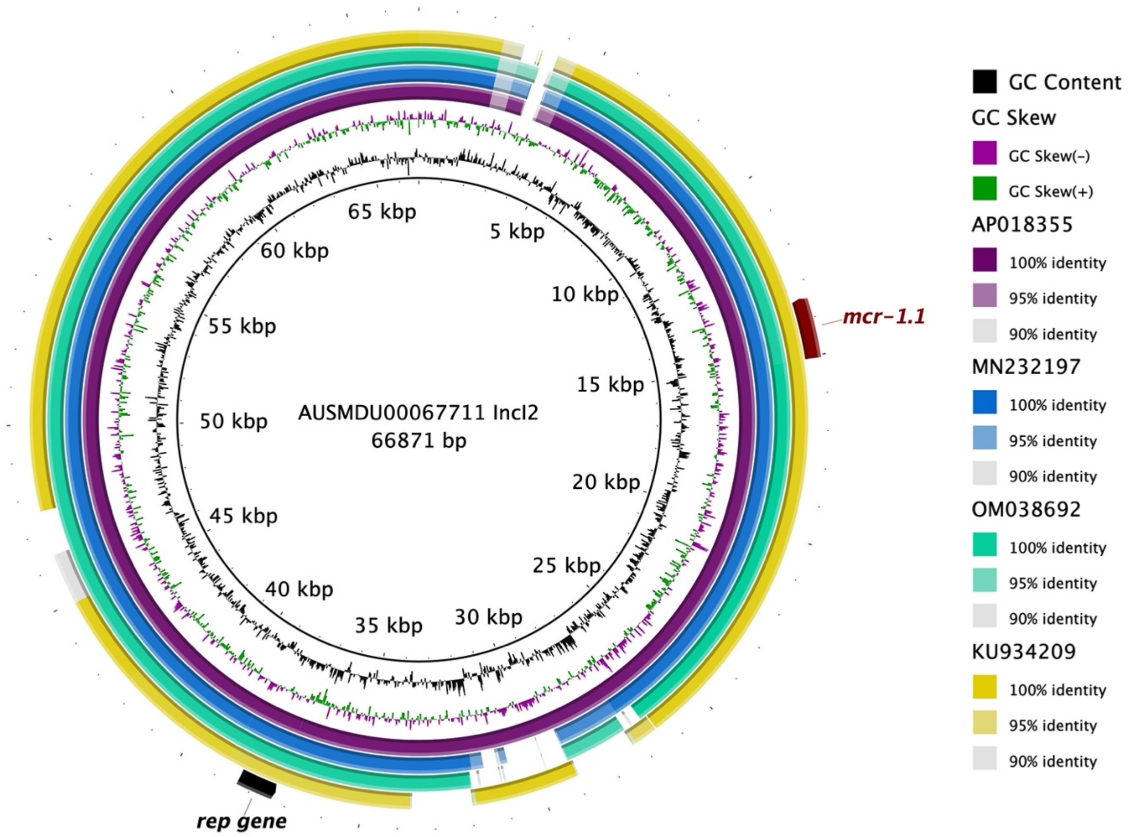

Supplementary Figure 4: Comparison of publicly available plasmids with IncI plasmid in AUSMDU00067711

BRIG plot demonstrating the alignment of the plasmid from the BAPS lineage 3 isolate AUSMDU00067711 harbouring the *mcr1.1* gene to public reference IncI2 plasmids from *E. coli* (Accessions: OM038692.1, MN232197.1, AP018355.1) and *S.* Albany (KU934209.1).
